# Characteristics of JYNNEOS Vaccine Recipients Before and During a Large Multiday LGBTQIA+ Festival — Louisiana, August 9–September 5, 2022

**DOI:** 10.15585/mmwr.mm7143e3

**Published:** 2022-10-28

**Authors:** Rieza H. Soelaeman, Lee Mendoza, Robert McDonald, Julie Hand, Theresa M. Sokol, Southern Decadence Preparedness and Response Team, Southern Decadence Preparedness and Response Team, John T. Brooks, Preetam Cholli, Maribeth Eckert, Braiden Eilers, Jennie J. Gayden, Craig Hayes, Irma Kocer, Sarah M. Labuda, Anna Llewellyn, Scott Santibañez, Colleen Scott, Megan Swanson, Arun Adhikari, Arundhati Bakshi, Samuel Burgess, Javone Davis, Jimmy Gale, Jenna Iberg Johnson, Suryatapa Kar, Kevin Litten, Katherine Murphy, Alyson Neel, Emma Ortega, Gillian Richardson, Sean Simonson, Yanling Zhao

**Affiliations:** ^1^CDC Monkeypox Emergency Response Team; ^2^Louisiana Department of Health.; CDC; CDC; CDC; CDC; CDC; CDC; CDC; CDC; CDC; CDC; CDC; CDC; Louisiana Department of Health; Louisiana Department of Health; Louisiana Department of Health; Louisiana Department of Health; Louisiana Department of Health; Louisiana Department of Health; Louisiana Department of Health; Louisiana Department of Health; Louisiana Department of Health; Louisiana Department of Health; Louisiana Department of Health; Louisiana Department of Health; Louisiana Department of Health; Louisiana Department of Health.

Since May 2022, 27,558 monkeypox cases have been identified in the United States (*1*). Gay, bisexual, and other men who have sex with men (MSM) represent the most affected demographic group in the current multinational outbreak (*2*). As of October 18, 2022, Louisiana had reported 273 monkeypox cases with 187 (68.5%) among residents of the Louisiana Department of Health (LDH) Southeast Region, which includes the city of New Orleans (*3*).

Southern Decadence is an annual multiday festival in New Orleans catering to persons who are lesbian, gay, bisexual, transgender, queer or questioning, intersex, asexual, and others (LGBTQIA+) that draws 150,000–300,000 participants annually. To prepare for the 2022 festival (September 1–5), LDH requested CDC collaboration for preparedness planning, including statewide pre-event administration of JYNNEOS vaccine to prevent monkeypox. CDC helped acquire 1,500 vials (≤6,000 intradermal doses) of JYNNEOS vaccine through a federal monkeypox large event vaccine pilot in addition to the state’s Phase 3 allocation of 4,400 doses.* Concurrently, LDH staff members used multiple strategies to reach out to the LGBTQIA+ community to provide education and increase vaccination coverage among eligible persons, based on local vaccination eligibility criteria (*3*).

Vaccination was available free of charge through public and private health clinics (clinics) beginning in July 2022.^†^ As part of the state’s prevention strategy to increase vaccination among eligible Louisiana residents in advance of Southern Decadence, LDH hosted a series of community vaccination events, including large volume events in central locations and smaller, more focused events. The smaller events, organized in collaboration with community partners, were aimed at removing barriers (e.g., stigma and mistrust) and reducing disparities by reaching populations at highest risk, especially persons with limited access to medical care. These community vaccination events were held across the state before and during the festival (August 9–September 5, 2022). Vaccination venues were purposefully selected at locations where MSM would feel comfortable seeking vaccination, such as gay-owned or gay-frequented venues. Patient demographic information was collected at the time of vaccination by the LDH Immunization Program. Aggregate data were shared with CDC. This activity was reviewed by CDC and was conducted consistent with applicable federal law and CDC policy.^§^

More than one half (58.6%) of Louisiana monkeypox cases have occurred among persons who identify as non-Hispanic Black or African American (Black) (*3*). Selection of community vaccination venues included a focus on gay bars and clubs with primarily Black patrons to help ensure more equitable access to vaccines. Community vaccination efforts continued during Southern Decadence and were held daily in a central venue (the Health Hub) three blocks from the main festival area. The Health Hub offered vaccines and testing for monkeypox and COVID-19, HIV screening, condoms, safe injection kits, fentanyl test strips, naloxone, and other health resources.

During August 9–September 5, 2022, a total of 6,854 doses of JYNNEOS were administered in Louisiana (Table), with 53.0%, 34.8%, and 12.2% administered at clinics, non–Health Hub community vaccination events, and the Health Hub, respectively.^¶^ Among persons who received vaccine outside the Health Hub, 90.1% were Louisiana residents; 54%, 24.0%, and 6.7% were non-Hispanic White (White), Black, and Hispanic or Latino (Hispanic) persons, respectively. Among Health Hub vaccine recipients, 45.5% were Louisiana residents, and 52.3%, 13.9%, and 10.3% were White, Black, and Hispanic persons, respectively. Residents of California, Florida, New York, and Texas accounted for 26.0% of Health Hub vaccine recipients.

**TABLE:** Characteristics of JYNNEOS vaccine recipients at health clinics,* community vaccination events,^†^ and at the Southern Decadence Health Hub^§^ (N = 6,854) — Louisiana, August 9–September 5, 2022

Characteristic	% of Vaccine administrations by vaccination setting, no. (column %)			
	Non–Southern Decadence Health Hub,^¶^ Aug 9–Sep 2			Southern Decadence Health Hub, Sep 1–5
	Health clinic	Community vaccination event	Total	
**Total (row %)**	**3,633 (53.0)**	**2,384 (34.8)**	**6,017 (87.8)**	**837 (12.2)**
**Sex**				
Male	3,145 (86.6)	2,003 (84.0)	**5,148 (85.6)**	697 (83.3)
Female	478 (13.2)	352 (14.8)	**830 (13.8)**	121 (14.5)
Other	6 (0.2)	20 (0.8)	**26 (0.4)**	18 (2.2)
Unknown	4 (0.1)	9 (0.4)	**13 (0.2)**	1 (0.1)
**Age group, yrs**				
0–17	15 (0.4)	0 (0.0)	**15 (0.2)**	3 (0.4)
18–29	631 (17.4)	455 (19.1)	**1,086 (18.1)**	108 (12.9)
30–49	1,878 (51.7)	1,228 (51.5)	**3,106 (51.6)**	429 (51.3)
50–64	832 (22.9)	535 (22.4)	**1,367 (22.7)**	233 (27.8)
≥65	277 (7.6)	166 (7.0)	**443 (7.4)**	64 (7.7)
Race and ethnicity**				
Black or African American, NH	929 (25.6)	516 (21.6)	**1,445 (24.0)**	116 (13.9)
White, NH	2,051 (56.5)	1,218 (51.1)	**3,269 (54.3)**	438 (52.3)
Other, NH	327 (9.0)	278 (11.7)	**605 (10.1)**	84 (10.0)
Hispanic or Latino	245 (6.7)	157 (6.6)	**402 (6.7)**	86 (10.3)
Unknown	81 (2.2)	215 (9.0)	**296 (4.9)**	113 (13.5)
**State of residence**				
Louisiana	3,373 (92.8)	2,045 (85.8)	**5,418 (90.1)**	381 (45.5)
Other	129 (3.6)	214 (9.0)	**343 (5.7)**	373 (44.6)
Unknown	131 (3.6)	125 (5.2)	**256 (4.3)**	83 (9.9)
**Dose**				
First	2,979 (82.0)	2,339 (98.1)	**5,318 (88.4)**	769 (91.9)
Second	654 (18.0)	45 (1.9)	**699 (11.6)**	68 (8.1)

**Abbreviation:** NH = non-Hispanic.

* Public or private health clinic offering monkeypox vaccine.

^†^ Vaccination event organized by Louisiana Department of Health at locations purposefully selected to improve vaccine equity, such as gay-owned or -frequented venues.

^§^ A pop-up venue centrally located within three blocks from the main festival area offering monkeypox and COVID-19 vaccines and testing, HIV screening, condoms, safe injection kits, fentanyl test strips, naloxone, and other health resources.

^¶^ Community vaccination events on September 1–2 included in this category occurred at venues outside the Health Hub.

** Other includes persons who identify as Native Hawaiian or other Pacific Islander, American Indian or Alaska Native, or multiracial, and persons who declined to report. Persons reporting Hispanic or Latino ethnicity could be of any race.

Achieving vaccine equity among communities disproportionately affected by the outbreak is critical to stopping the spread of *Monkeypox virus* (*4*). Large LGBTQIA+ gatherings that attract substantial media and social media attention, such as Southern Decadence, provide an opportunity to build vaccine demand among local members of the LGBTQIA+ community through outreach regarding the importance of vaccination, engagement of racial and ethnic minority groups, and stimulation of pre-event local vaccination efforts. Community-based monkeypox vaccination events can provide an accessible safe space for vaccination while minimizing judgment and stigma. Increased availability of vaccines ahead of the festival, including at clinics and community vaccination events, and during the festival at the Health Hub, increased overall vaccine access while reaching different demographic groups. Vaccinations at non–Health Hub settings more frequently reached Louisiana residents who identified as racial and ethnic minorities, whereas vaccination at the Health Hub increased reach to residents of other states. Although vaccination has not reached parity with racial and ethnic case distribution, the percentage of vaccine recipients who identified as Black at non–Health Hub settings (24.0%) was higher than that before August 9 (16.0%)** and at the Health Hub (13.9%). These data suggest that community engagement, targeted messaging, and selection of venues catering primarily to racial and ethnic minorities for community vaccination events can improve vaccine equity and reduce health disparities.

## Footnotes

* The Administration for Strategic Preparedness and Response and CDC used a phased distribution strategy to prioritize monkeypox vaccines for areas with higher numbers of cases. Jurisdictions began ordering Phase 3 allocations for vaccines to be distributed in August 2022 on July 29, 2022. https://aspr.hhs.gov/monkeypox/OperationalGuidance/Pages/distribution.aspx

† LDH maintains an updated list of public and private health clinics offering monkeypox vaccine and community vaccination events. https://ldh.la.gov/assets/oph/monkeypox/vaccine-locations/MonkeypoxVaccineLocations.pdf

§ 45 C.F.R. part 46, 21 C.F.R. part 56; 42 U.S.C. Sect. 241(d); 5 U.S.C. Sect. 552a; 44 U.S.C. Sect. 3501 et seq.

¶ Louisiana providers began using intradermal administration of JYNNEOS vaccine on August 10, 2022, after a Food and Drug Administration emergency use authorization for intradermal injection among persons aged ≥18 years who are determined to be at high risk for monkeypox to increase the total number of doses available for use up to fivefold on August 9, 2022. Subcutaneous administration was used at the first two community vaccination events on August 9 and August 11. Intradermal administration at community vaccination events began on August 13. https://www.fda.gov/news-events/press-announcements/monkeypox-update-fda-authorizes-emergency-use-jynneos-vaccine-increase-vaccine-supply

** Data from vaccine recipients during July 22–August 8, when JYNNEOS vaccine became available to Louisianans under expanded postexposure prophylaxis (1,106).
